# Targeted Albumin Infusions Do Not Improve Systemic Inflammation or Cardiovascular Function in Decompensated Cirrhosis

**DOI:** 10.14309/ctg.0000000000000476

**Published:** 2022-03-23

**Authors:** Louise China, Natalia Becares, Camilla Rhead, Thais Tittanegro, Nick Freemantle, Alastair O'Brien

**Affiliations:** 1 UCL Institute for Liver and Digestive Health, London, UK;; 2 Comprehensive Clinical Trials Unit, University College London, London, UK.

## Abstract

**INTRODUCTION:**

Albumin is recommended in decompensated cirrhosis, and studies have shown potential immunomodulatory effects. However, 2 large trials of repeated albumin infusions demonstrated contrasting results between outpatients and hospitalized patients. We investigated markers of systemic inflammation, immune function, albumin binding, and cardiovascular function using samples from Albumin To prevenT Infection in chronic liveR failurE (ATTIRE) taken at baseline, day 5, and day 10 of the trial to identify why targeted albumin infusions had no effect in hospitalized patients.

**METHODS:**

Plasma samples were analyzed from 143 patients (n = 71 targeted albumin; n = 72 standard care at baseline) for cytokines, cardiovascular markers, prostaglandin E_2_, the effect of plasma on macrophage function, and albumin radioligand binding and oxidation status. The sample size was based on our feasibility study, and samples were selected by a trial statistician stratified by the serum albumin level and the presence of infection at randomization and analyses performed blinded to the study arm. Data were linked to 3-month mortality and treatment groups compared.

**RESULTS:**

Increased baseline model for end-stage liver disease score, white cell count, calprotectin, CD163, tumor necrosis factor, renin, atrial natriuretic peptide, and syndecan-1 were associated with 3-month mortality. Despite infusing substantially differing volumes of albumin, there were no significant differences in inflammatory markers, albumin–prostaglandin E_2_ binding, or cardiovascular markers between treatment arms.

**DISCUSSION:**

Contrary to many preclinical studies, targeted intravenous albumin therapy in hospitalized decompensated cirrhosis had no effect across a broad range of systemic inflammation, albumin function, and cardiovascular mediators and biomarkers compared with standard care, consistent with the null clinical findings.

## INTRODUCTION

Many studies have shown that human albumin solution (HAS) infusions restore normovolemia and modify neurohumoral mechanisms in patients with cirrhosis with peripheral arterial vasodilation (1), supporting the hypothesis that infusions prevent cardiovascular and renal dysfunction in decompensated cirrhosis. Consequently, albumin infusions are recommended in all international guidelines following large volume paracentesis, diagnosis of spontaneous bacterial peritonitis and hepatorenal syndrome (2–4). We and others have also previously demonstrated potential immunomodulatory or anti-inflammatory effects of albumin infusions in hospitalized patients with complications of cirrhosis (5–10). In addition, there is growing interest in the association between circulating albumin dysfunction and poor clinical outcome in patients with decompensated cirrhosis (11,12) and whether treating patients with exogenous albumin infusions could improve this (10). Finally, the large-scale long-term albumin administration in decompensated cirrhosis (ANSWER) trial showed improved outcomes for patients treated with weekly albumin infusions, including a reduction in spontaneous bacterial peritonitis (SBP) and non-SBP infections (13).

In contrast, the Albumin To prevenT Infection in chronic liveR failurE (ATTIRE) trial demonstrated that targeted HAS therapy (a median of 200 g of albumin was infused achieving a serum albumin >30 g/L) had no effect on the incidence of infection, renal dysfunction, or mortality compared with standard care (a median of 20 g was given achieving a serum albumin of 25 g/L) (14). Subgroup analyses were also wholly neutral. Given the strength of the preclinical data and widespread use of albumin, this was surprising, and as patients with decompensated cirrhosis are frequently hospitalized, the differing results between this and the outpatient ANSWER studies are important. Therefore, we aimed to explore whether analyses of plasma samples taken during the ATTIRE trial could explain why targeted albumin infusions had no effect to guide future use or research into albumin in hospitalized patients with decompensated cirrhosis.

We investigated laboratory markers of systemic inflammation, immune function, albumin binding, infection, and cardiovascular/renal function using samples from ATTIRE patients taken at baseline (pretrial treatment), day 5, and day 10 of trial treatment. Day 5 was selected as most targeted albumin patients had achieved serum albumin >30 g/L at this stage, and patients who had been discharged or died before day 5 were not likely to benefit from albumin. We also examined the serum white cell count and C-reactive protein, which were measured on site. As multiple markers have demonstrated to be abnormal in patients with advanced liver disease compared with healthy volunteers, we sought to validate these markers by comparing levels in survivors and nonsurvivors at 3 months and then investigated the effect of targeted albumin infusions.

## METHODS

### Patients, intervention, consent, and definitions of clinical outcome

For full details of the ATTIRE study, please refer to the protocol paper (15) and randomized controlled trial (14). In brief, we conducted a randomized, multicenter, open-label, parallel-group trial involving hospitalized patients with decompensated cirrhosis who had a serum albumin level of <30 g per liter at enrollment. Patients were randomly assigned to receive either targeted 20% HAS for up to 14 days or until discharge, whichever came first, or standard care. Treatment commenced within 3 days of admission (see Supplementary Figure S1, http://links.lww.com/CTG/A783).

A sample of 143 patients from a total of 777 patients randomized had plasma samples blindly analyzed (n = 71 in the targeted HAS arm; n = 72 in the standard of care arm), see below for details on sample collection and selection. The trial was approved by the London–Brent Research Ethics Committee (ref: 15/LO/0104) and the Medicines and Healthcare Products Regulatory Agency (ref: 20363/0350/001-0001). Written informed consent was obtained from the patients. For incapacitated patients, a legal representative provided written informed consent until the patient regained capacity.

### Sample collection and processing

Blood samples were obtained from patients in both study arms at study recruitment (day 1), before treatment with albumin, and at days 5 and 10 after recruitment. This enabled us to study patients who had been in trial for at least 5 days, excluding patients who died early during hospitalization or were discharged before this point, as neither group would be likely to benefit from targeted albumin infusions. Samples were taken using 9 mL lithium heparin tubes that were labeled with the patient's trial ID and day of sample collection and transferred to the site's hospital laboratories and spun at ×1,300*g* at 20 °C. The plasma layer was removed and frozen at −80 °C in 2 mL cryovials with the corresponding trial identifier. Samples were collected from patients at 33 UK hospital sites. They were transferred to University College London at the end of the recruitment period in 2019. All analyses were conducted after the first sample thaw.

### Plasma prostaglandin E_2_ binding (^3^H-PGE_2_ equilibrium dialysis)

A Rapid Equilibrium Dialysis (RED) Device Single-Use Plate (Thermo Scientific, Waltham, MA) was used with 10 μL of prostaglandin E_2_ (PGE_2_)/3H-PGE_2_ (68.22 μM PGE_2_ and 125.2 cpm/pmol) was incubated with 240 μL HAS, plasma, or control for 30 minutes. Whenever possible, 3 technical repeats for each sample were obtained. Samples were dialyzed against phosphate-buffered saline in the red plate for 4 hours at 37 °C. For scintillation counting, 150 μL of sample to be counted was dissolved in 5 mL of scintillation fluid (EcoScint A; Science Laboratory Supplies, UK) in 20 mL polypropylene counting vials (Thermo Fisher Scientific, S31). Vials were shaken and then placed in racks within a counter. Reference ranges were taken before counting of the samples. Counts from the sample and buffer were then measured, and the percent bound was calculated using % Bound = 100–([cpm buffer chamber/cpm plasma chamber] × 100).

### PGE_2_ enzyme immunoassay

PGE_2_ concentration in plasma samples was determined using the Amersham PGE_2_ Biotrak Enzyme Immunoassay (EIA) System (GE Healthcare, Chicago, IL) as per the manufacturer's instructions. In brief, this assay relies on the forward sequential competitive binding technique whereby PGE_2_ in a sample competes with peroxidase-labelld PGE_2_ for a limited number of binding sites on a mouse monoclonal antibody. Samples were first lyzed to dissociate PGE_2_ from soluble receptors or interfering binding proteins in plasma, leaving total PGE_2_ to be analyzed. The sample and labeled PGE_2_ were added to the precoated wells absorbance simultaneously leading to direct competition for binding. After several washes, quantification of peroxidase-labeled PGE_2_ was performed by monitoring the enzymatic activity of peroxidase in the presence of the substrate 3,3′,5,5′-tetramethylbenzidine, which was measured spectrophotometrically by the increased absorbency at 450 nm. Therefore, absorbance intensity was inversely proportional to the PGE_2_ concentration in the sample. Unknown concentrations were determined via interpolation to a reference curve generated from a series of known PGE_2_ concentration assays. Our previous study has shown that although EIA measurements of PGE_2_ were as much as ×20 higher than liquid chromatography–tandem mass spectrometry, EIA reproducibly produced qualitative differences between sample groups consistent with data from liquid chromatography–tandem mass spectrometry analysis (16).

### R&D Systems Luminex Assay

Evaluation of 14 plasma cytokines, chemokines, and small proteins known to be involved with the inflammatory response and immune regulation was undertaken via Luminex assay (R&D Systems, Minneapolis, MN) according to the manufacturer's instructions. This is a bead-based multiplex assay allowing accurate, concurrent measurement of multiple analytes in a small volume of sample. Briefly, after defrosting, samples were centrifuged at 16,000*g* for 6 minutes and then diluted in calibrator diluent RD6-52 (1:2 for all analytes apart from sCD14 and lipopolysaccharide binding protein (LBP) in which assays plasma was diluted to 1:200). Standards were made up as per the specific product sheet and diluted 1:3 serially to produce a standard curve with the range of detection. Samples and standards were plated using the supplied opaque plate, and the microparticle cocktail was added as per the instruction. The plate was then sealed with foil and left overnight (14–16 hours) at 4 °C on an orbital shaker at 900 rpm. Plates were washed with the addition of a plate magnet, and antibody cocktail for the same analytes was added with the plate left on the orbital shaker at 900 rpm for 1 hour at room temperature. Plates were washed again, with the use of a magnetic plate, and streptavidin-phycoerythrin conjugate was added, and the plates were placed on the orbital shaker at 900 rpm for 30 minutes. The plate then underwent a final wash procedure, and the remaining particles were then resuspended in wash buffer, placed on the orbital shaker at 900 rpm for 5 minutes, and read on a Bio-Rad Bio-Plex reader to determine individual cytokine concentrations interpolated from a standard curve of known concentrations. Measured analytes with Luminex and range of detection are listed in the supplementary methods (see Supplementary Table S1, http://links.lww.com/CTG/A783).

### Lipopolysaccharide-stimulated monocyte-derived macrophage assay

Lipopolysaccharide (LPS)-stimulated monocyte-derived macrophage (MDM) assay consisted of 3 stages (10). Stage 1 consisted of *in vitro* differentiation of blood-borne healthy volunteer monocytes into macrophages. Stage 2 was LPS stimulation of the MDMs in the presence of patient plasma. Stage 3 was removal of supernatants and measurement of TNF using *in vitro* differentiation of blood-borne monocytes into macrophages. These are described in detail in the supplementary methods section (see Supplementary File, http://links.lww.com/CTG/A783).

### High-performance liquid chromatography analysis of plasma

Albumin was fractionated by high-performance liquid chromatography (HPLC) to give 3 peaks according to cysteine-34 in the free sulfhydryl form, mercaptalbumin (HMA), as a mixed disulphide, nonmercaptalbumin1 (HNA1), or in a higher oxidation state, nonmercaptalbumin2 (HNA2). Plasma was diluted 1:4 with sample buffer: 0.2 M dibasic sodium phosphate (49 parts) and 0.2 M monobasic sodium phosphate (51 parts) with 0.3 M NaCl with a pH of 6.8. All solvents and solutions were filtered through a filter unit (0.22 μm, Sterivex-GS; Millipore, Billerica, MA) before use. Ten microliters of the diluted plasma was injected into the HPLC system (AKTA pure; GE Healthcare Life Sciences) using a Shodex Asahipak ES-502N 7C anion exchange column (Showa Denko, Tokyo, Japan) and 0.2 M sodium acetate, 0.4 M sodium sulfate, pH 4.85 as mobile phase. For elution, a gradient of 0%–6% ethanol and a flow rate of 0.6 mL/min were used. The column was kept at room temperature. Detection was performed by fluorescence at 280/340 nm. The HPLC data were subjected to numerical curve fitting, and each albumin peak shape was approximated by a Gaussian function for calculation of the area under the peak. Quantification was based on peak heights determined by chromatography software (Unicorn 7.3 Evaluation Classic; GE Healthcare).

### Plasma calprotectin (measured at Gentian Laboratories, Sweden)

Plasma calprotectin levels were measured using the Gentian Calprotectin Turbidimetric Immunoassay GCAL (Gentian, Moss, Norway) in duplicate on a Cobas c501 analyzer (Roche, Basel, Switzerland). The samples were stored at −80 °C, and the assay was performed within 2 hours of thawing.

### Sample selection and statistical analysis

The minimum number of patient samples selected for analysis was based on previous measurements from the ATTIRE single-arm feasibility study (10). Based on the post-HAS treatment improvement in LPS-stimulated MDM TNFα production observed previously (17.7 ng/mL pretreatment vs 19.5 ng/mL posttreatment) with a known predicted sample size of 866 patients, a 2-sample paired means test (with a power of 0.8- and 2-sided *P* value of 0.05) required 47 patients in each treatment arm. Therefore, pretreatment and posttreatment planned analysis had a minimum of 94 patients.

After data entry of daily albumin levels at University College London (UCL) Comprehensive Clinical Trials Unit (CCTU), the trial statistician identified sample numbers for analysis corresponding to patients who had samples collected at days 1 and 5, with 50% of patients in the albumin treatment arm (achieving a serum albumin >30 g/L by day 5) and 50% of patients in the standard of care arm. The sample was stratified by the baseline albumin level (aiming to achieve a spread of starting albumins in the following groups: <20, 20–25, and 26–29 g/L) and the presence of infection at randomization. Samples available at day 10 for these patients were also used. A list of trial identification numbers was provided for analyses in pairs (2 samples for each patient). It was not known by the analyzer which treatment arm the patient was in, the baseline serum albumin, or any clinical information. After analyses were completed, all results were sent to a UCL CCTU trial statistician, and unblinding occurred 3 months after recruitment of the final patient, which represented the final follow-up period for the randomised controlled trial (RCT).

Statistical analysis used Graph Pad Prism 9.0. Unless stated, data are presented as mean ± SD. Two-tailed (unpaired) *t* tests were performed when comparing 2 independent groups of values with normal distribution. The Mann-Whitney *U* test was used for 2 independent groups of values when data were not normally distributed. The Wilcoxon rank-sum test was used to compare paired data that were not normally distributed. Although biomarkers/mediators tested were not normally distributed, and therefore data presented as median (interquartile range; Table 5), the differences between these biomarkers/mediators when comparing days were normally distributed and so these are presented as mean differences (Tables 3, 4, 6 and 7). This was considered the most appropriate method for analysis as baseline data (day 1) were similar in targeted albumin and standard care groups, as would be anticipated because these samples were taken prealbumin infusions. Furthermore, we include the confidence intervals (CI) for each analysis to enable additional scrutiny for these data. Given the multiple comparisons made, a prespecified *P* value of <0.01 was considered a significant finding. All authors had access to study data and reviewed and approved the final manuscript.

## RESULTS

### Baseline characteristics, albumin treatment, and clinical outcomes of patients

One hundred forty-three ATTIRE patients of the total 777 total were selected for blinded plasma analysis. Their baseline clinical characteristics were similar to the trial (14) (Table 1), with no differences between study arms. Patients' median age was 53.9 years, predominantly male, with alcohol as cirrhosis etiology. Most patients were admitted to hospital with complications of ascites; 28% were diagnosed with infection at baseline and 51% prescribed antibiotics. Targeted albumin treatment resulted in a sustained increment to serum albumin >30 g/L during the trial treatment period (see Supplementary Figure 2a, http://links.lww.com/CTG/A783) that was not observed in the standard care arm with substantially different amounts of albumin infused throughout the trial treatment period (see Supplementary Figure 2b¸ http://links.lww.com/CTG/A783).

**Table 1.** Baseline characteristics of the sampled patients

Baseline characteristics of the patients undergoing plasma analysis			
Characteristic	Albumin	Standard care	Total
	(N = 71)	(N = 72)	(N = 143)
Age, yr			
Median	53.4	55.3	53.9
IQR	12.7	13.7	14.0
Female sex, no. (%)	12 (16.9)	22 (30.5)	34 (23.8)
Etiology of cirrhosis, no. (%)			
Alcohol	63 (88.7)	60 (83.3)	123 (86.0)
Hepatitis C	5 (7.0)	8 (11.1)	13 (9.1)
NAFLD	5 (7.0)	10 (13.9)	15 (10.5)
Reasons for admission, no. (%)			
Encephalopathy	15 (21.1)	12 (16.7)	27 (18.9)
Suspected variceal bleed	8 (11.3)	6 (8.3)	14 (9.8)
Ascites management	48 (67.6)	45 (62.5)	93 (65.0)
Suspected infection	14 (19.7)	9 (12.5)	23 (16.1)
Jaundice	35 (49.3)	45 (62.5)	80 (55.9)
Serum albumin level, no. (%)			
<20 g/L	9 (12.7)	12 (16.7)	21 (14.7)
20–25 g/L	44 (62.0)	44 (61.1)	88 (61.5)
26–29 g/L	18 (25.4)	16 (22.2)	34 (23.8)
Physiological variable–median (IQR) long			
Creatinine (µmol/L)	65.0 (28.5)	71.5 (31.0)	68.0 (31.0)
Bilirubin (µmol/L)	106 (190.5)	102.5 (164.5)	103 (183.0)
MELD score–no. (%)			
<20	35 (49.3)	35 (48.6)	70 (49.0)
≥20	36 (50.7)	37 (51.4)	73 (51.0)
Diagnosed with infection, no. (%)	19 (26.8)	21 (29.2)	40 (28.0)
Prescribed antibiotics, no. (%)	39 (54.9)	34 (47.2)	73 (51.1)

IQR, interquartile range; MELD, model for end-stage liver disease; NAFLD, nonalcoholic fatty liver disease.

Clinical outcomes of the 143 patients undergoing sample analysis are described in Table 2. The incidence of renal dysfunction, infection, and death during the trial treatment period were similar to the main ATTIRE findings, with no difference between treatment arms, mortality at 28 days, 3 months, and 6 months.

**Table 2.** Clinical end points for patients undergoing plasma analysis

	Albumin (n = 71)	Standard care (n = 72)
Day 3–15		
Renal dysfunction	9 (12.7%)	14 (19.4%)
Infection	21 (29.6%)	17 (23.6%)
Death	4 (5.6%)	3 (4.2%)
Follow-up period		
Death at 28 d	7 (9.9%)	10 (13.9%)
Death at 3 mo	14 (19.7%)	17 (23.6%)
Death at 6 mo	22 (31.0%)	21 (29.2%)

### Targeted intravenous albumin infusions have no significant effect on plasma markers of inflammation, plasma-induced monocyte dysfunction, or albumin function in hospitalized patients with decompensated cirrhosis compared with standard care

Patients who died within 3 months of trial entry had significantly higher model for end-stage liver disease (MELD) score, serum white cell count, CD163, and TNFα at baseline compared with those who survived (Table 3). However, there were no significant differences between these markers when day 1 and 5 samples were compared from both treatment arms (Table 4). Neither were there any changes between days 1 and 10 (Table 5), although samples tested at this later time point were fewer in number as many patients had been discharged or died.

**Table 3.** Baseline plasma inflammatory profile in survivors and nonsurvivors at 3 months posttrial entry

	Survivors, n = 111	Nonsurvivors, n = 31	Difference	Confidence interval
MELD score	18.61	25.82	+7.21	3.90 to 10.51^a^
WCC (×10^9^/L)	8.6	10.8	+2.2	0.03 to 4.34^a^
CRP (mg/L)	39.2	47	+7.8	−16.19 to 31.78
sCD14 (ng/mL)	5,695	6,967	+1,272	−2,564 to 5,108
Procalcitonin (pg/mL)	402.2	456.3	+54.1	−289.7 to 398.0
LBP (ng/mL)	3,276	3,956	+ 680	−1,016 to 2,376
Calprotectin (mg/L)	0.9899	1.886	+0.8965	−0.3326 to 2.126
CD163 (ng/mL)	2,575	3,289	+714	172.7 to 1,255^a^
CCL8/MCP-2 (pg/mL)	54.45	57.29	+2.84	−9.685 to 15.36
IL1-β (pg/mL)	0.4899	0.8452	+0.3553	−0.1458 to 0.8563
IL-6 (pg/mL)	42.25	28.56	−13.68	−51.21 to 23.84
IL-10 (pg/mL)	2.12	12.94	+10.82	−13.85 to 35.49
TNF-α (pg/mL)	4.349	6.935	+2.587	0.0088 to 5.165^a^
IL-4 (pg/mL)	1.994	12.06	+10.07	−9.58 to 29.73
IL-8 (pg/mL)	199	277.6	+78.6	−37.12 to 194.4
PGE_2_ (pg/mL)	1,085	1,329	+244	−441 to 928.9
MDM TNFα (pg/mL) production in the presence of patient plasma	11,244	10,119	−1,125	−2,319 to 69.41
Albumin-PGE_2_% binding capacity	51.3	35.73	15.58	4.143 to 27.01^a^

Mean values were measured at baseline. Unpaired *t* test with Welch correction used to compare groups.

a Denotes significant difference *P* < 0.05.

CRP, C-reactive protein; MELD, model for end-stage liver disease; MDM, monocyte derived macrophage; PGE_2,_ prostaglandin E_2_; WCC, white cell count.

**Table 4.** Mean differences in inflammatory marker profile and albumin binding capacity between days 1 and 5 in targeted albumin and standard care patients

	Albumin (n = 71^a^)	Standard care (n = 72^a^)	Confidence interval (comparing treatment arm changes)
	Mean change, D1–D5	Mean change, D1–D5	
WCC (×10^9^/L)	−0.05	0.44	−1.149 to 2.134
CRP (mg/L)	−12.84	−9.57	−10.83 to 17.38
sCD14 (ng/mL)	979.5	812.7	−3,294 to 2,960
Procalcitonin (pg/mL)	4.439	−87.17	−462.5 to 279.2
LBP (ng/mL)	−522.2	−349.7	−753.1 to 1,098
Calprotectin (mg/L)	−0.064	−0.038	−0.3428 to 0.3934
CD163 (ng/mL)	−11.83	124.8	−188.6 to 461.8
CCL8/MCP-2 (pg/mL)	−0.7685	−5.076	−18.21 to 9.596
IL1-β (pg/mL)	0.048	0.006	−0.3225 to 0.02384
IL-6 (pg/mL)	8.422	−54.15	−131.2 to 6.093
IL-10 (pg/mL)	−0.8917	−1.163	−2.919 to 2.376
TNF-α (pg/mL)	−0.4076	−0.5263	−1.112 to 0.8747
IL-4 (pg/mL)	−0.1717	−0.6804	−2.412 to 1.395
IL-8 (pg/mL)	6.908	−72.81	−215.3 to 55.90
PGE_2_ (pg/mL)	−266.2	−86.88	−124.3 to 482.9
MDM TNFα (pg/mL) production in the presence of patient plasma	−219.5	151.3	−818.4 to 1,560
Albumin-PGE_2_ %binding capacity	10.10	8.33	−9.38 to 5.85

Unpaired *t* test with Welch's correction used to compare groups.

CRP, C-reactive protein; MDM, monocyte derived macrophage; PGE_2,_ prostaglandin E_2_; WCC, white cell count.

a Number of D1-D5 sample pairs ranged from 67 to 46.

**Table 5.** Median (IQR) values of all plasma markers tested at days 1, 5, and 10 of the study in targeted albumin and standard care patients

	Albumin			Standard care		
	Day 1	Day 5	Day 10	Day 1	Day 5	Day 10
	n = 69	n = 60	n = 35	n = 72	n = 51	n = 34
CRP (mg/mL)	29 (40)	19 (25.5)	19 (29)	25 (36.3)	24.5 (27)	22 (27.5)
WCC (×109/L)	8 (5.2)	8.2 (5.25)	9 (8.5)	7.6 (5.2)	7.2 (4.7)	10.4 (9.7)

	ln = 69	n = 67	n = 11	n = 70	n = 65	n = 9
MDM TNFα (pg/mL) production in the presence of patient plasma	11,690 (4,475)	11,355 (4,582)	9,969 (5,078)	11,367 (5,140)	10,803 (3,316)	10,942 (4,482)

	n = 71	n = 62	n = 15	n = 71	n = 57	n = 13
sCD14 (ng/mL)	2,970 (8,260)	4,840 (9,970)	1,070 (898.2)	2,230 (7,890)	4,150 (8,740)	944.4 (3,519)
Procalcitonin (pg/mL)	127.8 (283.9)	83.83 (237.9)	117.2 (102.6)	167.6 (213.2)	213.1 (287.2)	95.54 (199.2)
LBP (ng/mL)	1,780 (3,750)	1,495 (1,440)	6,150 (7,600)	2,080 (4,700)	1,540 (3,347)	2,850 (3,562)
Calprotectin (mg/L)	0.88 (0.885)	0.78 (0.925)	1.23 (1.22)	0.605 (1.048)	0.76 (0.65)	0.65 (0.71)
CD163 (ng/mL)	2,531 (2,074)	2,622 (2,160)	1,969 (2,203)	2,616 (1,536)	2,515 (1,779)	2,986 (2,508)
CCL8/MCP-2 (pg/mL)	44.95 (43.13)	45.98 (41.34)	35.3 (30.9)	50.2 (27.55)	48.32 (26.79)	45.29 (35.54)
IL1-β (pg/mL)	0 (1.3)	0 (1.33)	0 (0)	0 (1.3)	0 (1.33)	0 (0.12)
IL-6 (pg/mL)	10.4 (13.8)	9.74 (9.55)	17.26 (15.42)	14.5 (23.3)	8.6 (15.19)	7.63 (6.54)
IL-10 (pg/mL)	0 (0.35)	0 (0.55)	0 (0.67)	0 (0.9)	0 (1.29)	0 (1.82)
TNF-α (pg/mL)	3.6 (3.9)	3.61 (4.23)	2.89 (4.34)	4.2 (3.1)	4.02 (2.68)	2.88 (2.46)
IL-4 (pg/mL)	0 (0)	0 (0)	0 (4.9)	0 (4.2)	0 (4.19)	0 (0)
IL-8 (pg/mL)	68.3 (332.1)	45.18 (156.3)	67.43 (185.5)	76.9 (201.1)	63.9 (148.7)	19.94 (144.3)
PGE_2_ (pg/mL)	716.1 (768.4)	649.6 (550.7)	625.1 (756.8)	726.6 (696.3)	632.1 (626.7)	765.4 (1,352)
Renin (pg/mL)	754.1 (1,328)	644.2 (1,026)	298.2 (1,226)	930.8 (1,500)	869.7 (1,117)	736.4 (1,347.5)
Syndecan-1 (pg/mL)	2,084 (764)	1,984 (780)	1,904 (961)	2,122 (683)	2,105 (560)	2,063 (333)
ANP (pg/mL)	8,380 (7,539)	8,503 (7,491)	9,322 (5,944)	8,529 (7,512)	8,129 (4,711)	5,424 (5,412)

ANP, atrial natriuretic peptide; CRP, C-reactive protein; MDM, monocyte-derived macrophage; PGE_2,_ prostaglandin E_2_; WCC, white cell count.

To examine patients with likely infection at baseline, those treated with antibiotics at randomization were analyzed separately (Table 6). IL-6 fell significantly in standard care patients, but no other biomarkers differed between groups.

**Table 6.** Mean differences in inflammatory marker profile and albumin binding capacity between days 1 and 5 in targeted albumin and standard care patients who were prescribed antibiotics at baseline

	Albumin (n = 37^a^)	Standard care (n = 34^a^)	Confidence interval (comparing treatment arm changes)
	Mean change, D1–D5	Mean change, D1–D5	
WCC (×10^9^/L)	−0.83	−0.61	−1.84 to −0.2.27
CRP (mg/L)	−22.66	−16.88	−17.98 to 29.54
sCD14 (ng/mL)	2,555	866.6	−7,092 to 3,715
Procalcitonin (pg/mL)	33.92	253.4	−320.1 to 759.2
LBP (ng/mL)	−648.3	−407.8	−1,293 to 1,774
Calprotectin (mg/L)	−0.11	−0.01	−0.45 to 0.67
CD163 (ng/mL)	63.32	−24.24	−586.9 to 411.8
CCL8/MCP-2 (pg/mL)	4.499	−15.31	−44.28 to 4.67
IL1-β (pg/mL)	0.05	0.10	−0.46 to 0.56
IL-6 (pg/mL)	0.91	−53.70	−108.6 to −0.59^b^
IL-10 (pg/mL)	−1.69	−0.79	−3.51 to 5.30
TNF-α (pg/mL)	−0.63	−1.10	−2.08 to 1.13
IL-4 (pg/mL)	1.04	−1.42	−5.65 to 0.73
IL-8 (pg/mL)	74.90	−40.39	−353.7 to 123
MDM TNFα (pg/mL) production in the presence of patient plasma	−279.4	−157.9	−1,563 to 1,806
PGE_2_ (pg/mL)	−126.2	−17.16	−272.2 to 490.3
Albumin-PGE_2_ %binding capacity	7.66	5.84	−8.23 to 4.60

Unpaired *t* test with used to compare groups.

CRP, C-reactive protein; MDM, monocyte-derived macrophage; PGE_2,_ prostaglandin E_2_; WCC, white cell count.

a Total of 36 pairs available for binding capacity analysis.

b Denotes significant difference *P* < 0.05.

We investigated the impact of patient plasma on monocyte function, having shown this to cause significant dysfunction previously (9,10). Plasma from patients who died within 3 months reduced healthy monocyte-derived macrophage LPS-induced TNFα production when compared with plasma from those who survived, but this did not reach significance (Table 3). There were no differences when patients who went on to develop nosocomial infection during the trial treatment period were compared with those that did not (see Supplementary Figure 3a, http://links.lww.com/CTG/A783), nor any differences between paired sample analysis from days 1 and 5 in both the albumin treated and standard care patients (see Supplementary Figure S3b, http://links.lww.com/CTG/A783).

We assessed albumin functionality using albumin PGE_2_-binding capacity of plasma samples as previously (10), which was significantly worse at baseline in patients that died within 3 months of trial recruitment compared with survivors (Table 3). Albumin binding capacity was 51.3% in survivors vs 35.73% in nonsurvivors (15.58% higher, *P* = 0.0082, CI 4.14%–27.01%). Patient plasma albumin binding capacity improved in both targeted albumin and standard care patients when day 1 and 5 samples were compared, with no differences in the magnitude of improvement between arms (Table 4; see Supplementary Figure 2c, http://links.lww.com/CTG/A783). In patients with infection at baseline, plasma albumin binding capacity improved in both targeted albumin and standard care patients when day 1 and 5 samples were compared, with no differences in magnitude of improvement (Table 6). A second assay to evaluate the presence of damaged, oxidized albumin similarly showed a trend to improvement in nonoxidized human mercaptalbumin (HMA) at day 5 in both groups in a small number of samples, but numbers were insufficient to detect a statistically significant difference (see Supplementary Figure 2d, http://links.lww.com/CTG/A783).

### Targeted intravenous albumin infusions have no significant effect on cardiovascular function markers in hospitalized patients with decompensated cirrhosis compared with standard care

**Figure 1.**
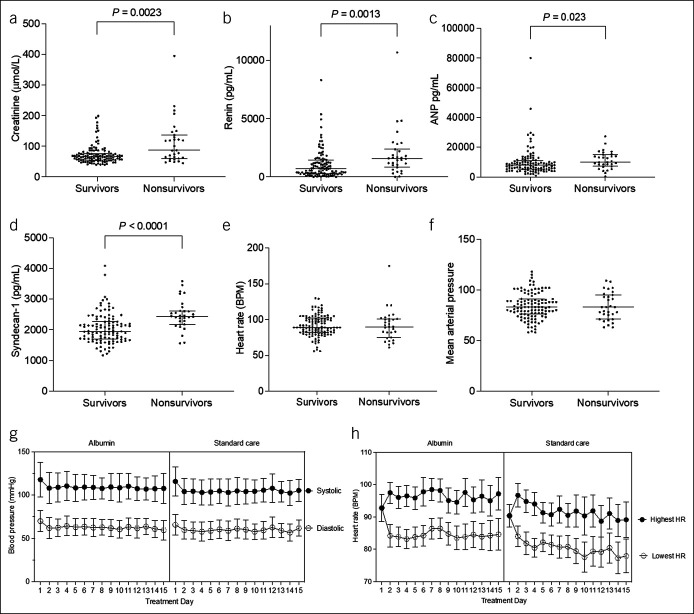
Baseline markers of circulatory and renal function in survivors vs nonsurvivors at 3 months after trial entry. (**a**) Creatinine in nonsurvivors (n = 31) and survivors (n = 112). (**b**) Renin in nonsurvivors (n = 31) and survivors (n = 108). (**c**) Atrial natriuretic peptide (ANP) in nonsurvivors (n = 31) and survivors (n = 108). (**d**) Syndecan-1 in nonsurvivors (n = 31) and survivors (n = 108). (**e**) Heart rate in nonsurvivors (n = 31) and survivors (n = 111). (**f**) Mean arterial pressure (MAP) in nonsurvivors (n = 31) and survivors (n = 111). Data presented as median values with Mann-Whitney statistical testing between groups, *P* values on figures. (**g**) Lowest daily paired systolic blood pressure recordings (with paired diastolic pressure) during the trial treatment period (mean and SD) for targeted albumin (n = 71) and standard care (n = 72) patients. (**h**) Heart rate variation during the trial treatment period (mean, SD) in targeted albumin (n = 71) and standard care (n = 72) patients.

Creatinine, renin, atrial natriuretic peptide (ANP), and syndecan-1 were all significantly higher at baseline in those patients died within 3 months of study recruitment (Figure 1a–d). Heart rate and mean arterial pressure at baseline did not differ between survivors or nonsurvivors at 3 months (Figure 1e–f), and variation in blood pressure and heart rate throughout the trial was similar in both treatment arms (Figure 1g–h). Albumin treatment had no overall significant impact on plasma renin, ANP, or syndecan-1 after 5 days of treatment compared with standard care (Table 7). As is in the main ATTIRE findings, there was a decrease in creatinine in both arms, with no difference in the magnitude between arms (−4.9 vs −5.1 μmol/L, CI −18.6 to 18.3 μmol/L).

**Table 7.** Mean differences in cardiovascular markers tested between days 1 and 5 in targeted albumin and standard care patients

	Albumin (n = 71^a^)	Standard care (n = 72^a^)	Confidence interval (comparing treatment arm changes)
	Mean change, D1–D5	Mean change, D1–D5	
Renin	−630.9	−18.32	−96.93 to 1,322
Syndecan-1	−88.73	487.3	−358.4 to 1,511
ANP	−78.04	−518.8	−2,329 to 1,447

Unpaired *t* test.

ANP, atrial natriuretic peptide.

a Number of D1-D5 sample pairs ranged from 67 to 46.

## DISCUSSION

We assessed a large number of samples from our completed RCT with patients selected using a predefined, appropriately powered method by the study sponsor (UCL CCTU) for experimental analyses, which were performed blinded to the trial arm or outcome. We demonstrate that albumin infusions to increase serum albumin >30 g/L in hospitalized patients with decompensated cirrhosis had no immunomodulatory or anti-inflammatory effect, nor improved circulating albumin function over standard care, in which little albumin was administered. When patients treated with antibiotics at baseline were analyzed separately, we also found no immunomodulatory or anti-inflammatory effect of targeted albumin infusions over standard care. Targeted albumin infusions did not significantly reduce plasma renin activity, nor improve markers of excess cardiac filling, heart rate variability, or blood pressure. These data are consistent with no differences seen between study arms of the ATTIRE RCT for development of new infection, renal dysfunction, nor mortality in the main trial.

These results differ from our feasibility trial article, in which albumin infusions appeared to have an immune effect (10). However, the feasibility samples were from a single-arm trial in which all patients received targeted albumin, comparing samples from days 1 and 3. We assume that the improvement in assay results observed reflected overall patient improvement between days 1 and 3 related to general clinical care, rather than an effect of albumin. In contrast, our current data were from 2 trial arms over an identical time frame. Our previous laboratory study that demonstrated albumin's ability to improve immune dysfunction by binding PGE_2_ (9) added albumin to macrophages in culture which would not replicate the effects of circulating oxidative stress on albumin function that exist in patients with decompensated cirrhosis (9). Circulating albumin is damaged in many severe disease settings (17), which alters its function (18) and may directly induce systemic inflammation leading to a suppressed immune response in decompensated cirrhosis (19). Consistent with other albumin functional studies in cirrhosis, we confirm that low binding capacity levels, as assessed by albumin-PGE_2_, correlated strongly with 3-month mortality (11,12). However, importantly, we demonstrate that targeted albumin infusions have no effect on circulating albumin function compared with standard care. Many of albumin's proposed immunomodulatory effects are linked to its ability to bind pathophysiological ligands (20), and we suggest that the lack of any effect of targeted albumin on infection outcome is because of its inability to improve circulating albumin binding capacity. In particular, we must conclude that albumin infusions do not reverse the effects of PGE_2_ *in vivo*. These findings may also, in part, explain the differences in infection outcomes between ANSWER and ATTIRE, in that albumin infusions may improve binding capacity in these outpatients as they most likely had less albumin dysfunction, with median MELD score 12–13, and alcohol abstinence at trial entry in ANSWER compared with an MELD of 20 and most patients with alcohol-induced cirrhosis in ATTIRE.

ATTIRE demonstrated no benefit in renal dysfunction or survival in the targeted albumin arm despite substantial differences in amount of albumin infused, and there was increased pulmonary edema. We therefore measured plasma levels of renin, NT-pro ANP, the cleaved N-terminal of ANP, and syndecan-1 to assess the impact of albumin on cardiovascular function. Increased plasma renin activity in decompensated cirrhosis reflects reduced effective blood or extracellular fluid volume, consequent to portal hypertension and mesenteric vasodilation, with reduction described following albumin infusions for large-volume paracentesis and SBP (6,21–23). Cardiac dysfunction is increasingly recognized in cirrhosis, especially those with alcohol etiology. ANP is released by atrial myocytes in response to distension and high levels occur during hypervolemic states, such as heart failure, and syndecan-1 putatively links systemic inflammation and sepsis with excessive cardiac volume loading (24). Elevated levels of all 3 markers at baseline strongly correlated with poor outcome in patients studied, supporting the coexistence of cardiac failure and hypovolemia in patients who died within 3 months, and targeted albumin had no significant impact on any of these. The future use of noninvasive methods to assess fluid volume and cardiac function to guide precision fluid resuscitation in these patients may lead to improved outcomes (25).

Our study has several limitations. Because of the nature of a 35-site clinical trial, we were only able to collect plasma samples at certain time points. Therefore, we did not evaluate wider phenotypic changes of the cellular immune response. Samples from day 10 were smaller in number and from patients who remained in the study for ongoing hospital treatment (they had not died, nor improved sufficiently for discharge) and therefore may not be representative of the entire cohort. Multiple statistical comparisons have been made, which must be considered when interpreting any positive *P* values; we have presented data with their CIs. There are several methods that can assess albumin function; we used PGE_2_ to assess albumin-ligand binding at Sudlow site II, as previously, which is decreased when albumin is modified posttranscription (11,26), and our findings were corroborated by HPLC albumin analysis. In contrast to the cardiovascular biomarkers, the lack of correlation with clinical outcomes for most inflammation markers tested questions their significance. It is possible that many simply reflect disease severity rather than true therapeutic targets. Better human experimental models of systemic inflammation and immune function linked to robust clinical endpoints may be required to develop effective immune therapies to prevent infection in these patients. Finally, most patients had alcohol-induced cirrhosis, and findings may differ for other causes of liver cirrhosis.

In conclusion, contrary to many preclinical studies, targeted intravenous albumin therapy in hospitalized patients with decompensated cirrhosis had no effect across a broad range of markers of systemic inflammation, albumin binding and oxidation, and cardiovascular function using samples from our large prospective, completed randomized and appropriately powered trial. This was entirely consistent with no clinical benefit seen. It seems that albumin infusions are unable to overcome the functional changes in circulating albumin caused by decompensated cirrhosis and that precision fluid resuscitation may be required to treat the complex cardiovascular phenotype of these patients.

## CONFLICTS OF INTEREST

**Guarantor of the article:** Alastair O'Brien, MD, PhD.

**Specific author contributions:** L.C. performed most analyses assisted by T.T., N.B., and C.R. A.O. and L.C. conceived the study and drafted the manuscript. N.F. provided substantial statistical support. All authors approved the final submitted draft.

**Financial support:** Funded by the Health Innovation Challenge fund awarded to A.O. (Wellcome Trust and Department of Health and Social Care) HICF reference HICF-R8-439, WT grant number WT102568.

**Potential competing interests:** None to report.

**Study Highlights**

**WHAT IS KNOWN**

✓ Albumin infusions are widely considered to prevent cardiovascular and renal dysfunction and reduce systemic inflammation in decompensated cirrhosis.

**WHAT IS NEW HERE**

✓ Contrary to previous studies, our analyses of a large number of samples from our completed clinical trial, targeted intravenous albumin therapy in hospitalized decompensated cirrhosis had no effect across a broad range of systemic inflammation, albumin function and cardiovascular mediators and biomarkers compared with standard care.

## Supplementary Material

SUPPLEMENTARY MATERIAL
