# Supplementary material for: Targeted Albumin Infusions Do Not Improve Systemic Inflammation or Cardiovascular Function in Decompensated Cirrhosis
Source: Clin Transl Gastroenterol. 2022 Mar 23;13(5):e00476. doi: 10.14309/ctg.0000000000000476 (PMC9132514; doi:10.14309/ctg.0000000000000476)
Supplement: SUPPLEMENTARY MATERIAL [file ct9-13-e00476-s001.docx]

## ATTIRE Trial Investigators

Independent Data Monitoring Group: Professor Dominique Valla (CHAIR), Tim Clayton and Professor Vipul Jairath.

Data centre at University College Comprehensive Clinical Trials Unit (UCL CCTU): Kate Bennett, Scott Bevanǁ, James Blackstone, Kashfia Chowdhury, Zainib Shabir and Simon Skene^

Trial Steering Committee: Professor Stephen J. Brett (CHAIR), John Crookenden (Patient Representative), Professor Shahid A. Khan, Brennan Kahan, Professor Graeme Alexander, Professor Humphrey Hodgson and Professor Mike Murphy.

Trial Management Group: Dr Louise China, Dr Ewan H Forrest, Dr Yiannis Kallis, Jim Portal, Professor Stephen Ryder and Dr Gavin Wright.

UCL CCTU: Dr Ana Arbeloa del Moral, James Blackstone, Kashfia Chowdhury, Dr Ana Carolina Estevao, Rosie Hamilton, Ms Khadra Mohamoud and Dr Nicola Muirhead.

Research Steering Committee: Professor Mauro Bernardi (CHAIR), Paula Milton (Department of Health and Social Care representative) and Nicola Shepherd (Wellcome Trust representative).

Microbiology and Adverse Event Review Panel: Dr Indran Balakrishnan, Dr Mark McPhail, Dr Brian Hogan and Dr Jane Abbott.

**ATTIRE Site Investigators**

Professor Aftab Ala, Dr Richard Aspinall, Dr Andrew Austin, Dr C Lye Ch'ng, Dr Jeremy Cobbold, Dr Lynsey Corless, Dr Alexandra Daley, Professor Matthew Cramp, Dr Ahmed Elsharkawy, Dr Alex Evans, Prof Graham Foster, Dr Shaun Greer, Dr Mathis Heydtmann, Dr Coral Hollywood, Dr Peter Isaacs, Professor Rajiv Jalan, Dr Richard Keld, Dr Andrew King, Dr Stuart McPherson, Dr Judith Morris, Professor Jane Metcalf, Dr Richard Parker, Dr Janisha Patel, Dr Francisco Porraz-Perez, Dr Praveen Rajasekhar, Dr John Ramage, Dr Paul Richardson, Dr Dariush Sadigh, Dr Deepak Suri, Dr Esther Unit, Professor Sumita Verma and Dr Earl Williams.

**ATTIRE Clinical Trial Sites**

Basildon, Basingstoke, Berkshire, Birmingham, Blackpool, Bournemouth, Bristol, Brighton, Coventry, Derby, Durham, Glasgow RI, Glasgow QE, Glasgow RA, Gloucestershire, Heartlands, Hull, Leeds, Liverpool, Manchester, Newcastle, North Tees, North Tyneside, Nottingham, Oxford, Plymouth, Portsmouth, Royal Free, Royal London, South Tyneside, Southampton, Surrey, Swansea, Whittington and Wigan.

## Supplementary methods: LPS stimulated monocyte derived macrophage assay

This consisted of three stages. Stage 1 was in vitro differentiation of blood-borne healthy volunteer monocytes into macrophages – monocyte derived macrophages (MDMs). Stage 2 was LPS stimulation of the MDMs in the presence of patient plasma. Stage 3 was removal of supernatants and measurement of Tumor Necrosis Factor (TNFα) using ELISA. Each stage is described below:

Stage 1: In vitro differentiation of blood-borne monocytes into macrophages

Isolation of monocytes from cones obtained from the NHS plateletpheresis service:

Due to donor-donor monocyte variation and the amount of MDMs required for these analyses, we used pooled white cells in leukoreduction system chambers that were obtained from the NHS blood donation service, Collingdale. These were from anonymous healthy platelet donors (plateletpheresis), these cones contained a concentrated proportion of white cells obtained during the plateletpheresis process (approximately 10-15x that which we obtained from 110mL of donated blood). LPS stimulation of MDMs sourced in this way, prior to the analysis, showed that the resulting TNFα production was comparable to the cells isolated locally. Approximately 10mL of concentrated cells were provided from one platelet donor. This volume was diluted up to 150mL Hanks Balanced Salt Solution (HBSS) divided into 3 falcons. 25mL of this dilution was then layered over 15mL of Ficoll Paque (6 falcons) and spun at 1000x *g*, 30 min, 25^o^C, brake off, low acceleration. The interface layer containing the monocytes was removed and placed with 2mL of ACK (Ammonium-Chloride-Potassium) lysis buffer per falcon (6 tubes). Cells were then washed with HBSS and counted. EasySep^TM^ negative selection human monocyte isolation kit (Stemcell, France) was used to isolate the monocytes from this stage (rosette sep could not be used as there were an inadequate number of red cells compared to the very high number of white cells present for the unwanted white cells to bind to). EasySep^TM^ labels unwanted cells (non-monocytes and CD16+ monocytes) with a magnetic isolation cocktail and a magnet is subsequently used to retain unwanted cells whilst monocytes are poured into a separate falcon for use. The protocol was used as per manufacturer’s instructions. After cells were counted, 100μl of isolation cocktail was added to 10 x10^7^ cells in 2mL HBSS and left at room temperature for 5 minutes. 100μl of magnetic particles were then added and again left for 5 minutes at room temperature prior to the total volume of the sample being topped up to 2.5mL and placed in the magnet for 2.5minutes. The enriched cell suspension containing CD14+ monocytes was subsequently removed and placed in a new falcon.

Culture of monocyte derived macrophages:

After isolation monocytes (either from a cone or direct blood donor) were counted and then re-suspended at 4x10^6^ cells/3mLs media in polystyrene plates (Corning^®^Costar^®^) and placed in an incubator at 37°C, 5% CO_2_. After one hour media with any non-adherent cells was removed and replaced with fresh media, which was then supplemented with 20ng/mL of M-CSF. After 3 days media was changed and re supplemented with 20ng/mL Macrophage colony-stimulating factor (M-CSF).

On day 6 media was aspirated and 1mL of lifting buffer (PBS plus 10mM EDTA and 4mg/mL lidocaine) at 10°C was added to each well and left for 20 minutes. Wells were then scraped and suspended cells removed within the lifting buffer and placed in a 50mL falcon which was topped up to 50mLs with PBS and spun at 300x *g* at 20°C for 5 minutes. The supernatant was again removed, and pellet was washed once more in 30mLs PBS and centrifuged at 300x *g* at 20°C for 10 minutes. The pellet was then resuspended in 1mL of media and cells were counted and then plated in a 96 well tissue culture treated plate (Corning^®^Costar^®^) at 50,000 cells/well in 100μl of media containing 20ng/mL M-CSF. Plates were incubated for 24 hours prior to experiments to allow cells to re-adhere.

Stage 2: LPS Stimulation

MDMs were treated sequentially as follows:

1. 50μl (25% v/v) healthy volunteer or patient plasma was added to each well

2. 50μl Lipopolysaccharide 200ng/mL (LPS; *Salmonella abortus equi* S-form*,* [TLR*grade™*], Enzo Life Science, 1ng/mL) *(end well concentration was 100ng/mL)*

After addition of LPS, cells were incubated for 4 hours (37°C/5% CO_2_) and 50μl of supernatant removed and stored at -80°C prior to analysis.

Due to well – well variation in this assay all samples were evaluated with 3 technical repeats and the mean of technical repeats was reported in the results section.

Stage 3. Single-Analyte Enzyme Linked Immunosorbent Assay

The concentration of TNFα in cell culture supernatants was measured via enzyme-linked immunosorbent assay (ELISA). Pre-validated kits employing the ‘sandwich’ principle of analyte-specific capture and biotinylated detection antibodies were obtained from R&D systems (USA, Duoset system) for the evaluation of analytes and conducted in half-volume (50μL) 96 well Corning CoStar high-binding, clear flat bottom polystyrene plates. Light absorbance of the streptavidin-horse radish peroxidase (HRP) catalysed breakdown of 3,3’,5,5’-tetramethylbenzidine (TMB) was measured at 450nM against a reference wavelength of 595nM on a Tecan® GENios™ microplate spectrofluorometer and sample values interpolated from a standard curve of known antigen concentration on a plate-by-plate basis. Supernatants and plasma samples were thoroughly thawed and diluted in reagent diluent (PBS containing 5% bovine serum albumin) prior to addition to ensure working concentrations in the centre of the standard curve (1:4 MM6, 1:40 MDM) and the HRP-TMB reaction stopped via the addition of 1M sulphuric acid.

## Figure S1. ATTIRE patient recruitment and treatment protocol

**Figure S2**. **(a)** Daily mean serum albumin values in patients from targeted albumin (albumin, n=71) and standard care arms (n=72) during the trial treatment period. Data presented as means with confidence intervals. **(b)** Total mean daily amount of albumin (g) infused in trial patients. **(c)** Patient plasma albumin binding capacity improved by day 5 in both albumin arm patients (median improvement of 5.9%, p<0.0001, n-42 paired samples) and standard care patients (median improvement of 3.7%, p=0.004, n=36 paired samples). *Wilcoxon test.* **(d)** There was a trend to improvement in the amount of non-oxidized human-mercapto-albumin (HMA) at day 5 in albumin treated patients (n=8) and in standard care patients (n=4).

**Figure S3**. **(a)** Baseline plasma mediated monocyte derived macrophage (MDM) TNFα production in patients who went onto develop infection (n=37) versus those who did not (n=102). TNFα was measured in supernatants 4 hours after MDMs had been stimulated with LPS in the presence of patient plasma. *Mann-whitney test.*

**(b)** Change in plasma mediated MDM TNFα production between days 1 and 5 in patients that developed a new infection during the trial treatment period, standard care (n=14 paired samples) versus albumin arm (n=20 paired samples). There were no changes in both arms (paired t-test).

| **Cytokine** | *Detection Range*  *(pg/mL)* | **Marker of vascular filling** | *Detection Range*  *(pg/mL)* | **Proteins elevated in acute inflammatory response** | *Detection Range*  *(pg/mL)* |
| --- | --- | --- | --- | --- | --- |
| IL-1β | 7,900 - 10.8 | NT-Pro Atrial natriuretic peptide | 129,860 - 178 | LPS binding protein | 32,990,000 - 45,254 |
| IL-6 | 1,460 - 2.0 | Syndecan-1 | 126,840 - 174 | Pro calcitonin | 4,160 - 5.7 |
| IL-8 | 1,440 - 2.0 | Renin | 52,660 - 72.2 | Soluble CD14 | 11,344,000 - 15,561 |
| IL-10 | 1,800 - 2.5 |  |  | CD163 | 2,648,800 - 3,633 |
| TNFα | 4,100 - 5.6 |  |  |  |  |
| IL-4 | 6,760 - 9.3 |  |  |  |  |
| CCL8/  MCP-2 | 6,800 -  9.3 |  |  |  |  |

**Table S1:** R&D Systems Luminex® Assay- Measured analytes with luminex and range of detection
